# Isolation of biologically active nanomaterial (inclusion bodies) from bacterial cells

**DOI:** 10.1186/1475-2859-9-66

**Published:** 2010-09-10

**Authors:** Špela Peternel, Radovan Komel

**Affiliations:** 1 Laboratory for Biosynthesis and Biotransformation, National Institute of Chemistry, Ljubljana, Slovenia

## Abstract

**Background:**

In recent years bacterial inclusion bodies (IBs) were recognised as highly pure deposits of active proteins inside bacterial cells. Such active nanoparticles are very interesting for further downstream protein isolation, as well as for many other applications in nanomedicine, cosmetic, chemical and pharmaceutical industry.

To prepare large quantities of a high quality product, the whole bioprocess has to be optimised. This includes not only the cultivation of the bacterial culture, but also the isolation step itself, which can be of critical importance for the production process.

To determine the most appropriate method for the isolation of biologically active nanoparticles, three methods for bacterial cell disruption were analyzed.

**Results:**

In this study, enzymatic lysis and two mechanical methods, high-pressure homogenization and sonication, were compared.

During enzymatic lysis the enzyme lysozyme was found to attach to the surface of IBs, and it could not be removed by simple washing. As this represents an additional impurity in the engineered nanoparticles, we concluded that enzymatic lysis is not the most suitable method for IBs isolation.

During sonication proteins are released (lost) from the surface of IBs and thus the surface of IBs appears more porous when compared to the other two methods. We also found that the acoustic output power needed to isolate the IBs from bacterial cells actually damages proteins structures, thereby causing a reduction in biological activity.

High-pressure homogenization also caused some damage to IBs, however the protein loss from the IBs was negligible. Furthermore, homogenization had no side-effects on protein biological activity.

**Conclusions:**

The study shows that among the three methods tested, homogenization is the most appropriate method for the isolation of active nanoparticles from bacterial cells.

## Background

In recent years, the rapid expansion of biotechnology has lead to the production of a wide spectrum of recombinant proteins. To this end, a range of host organisms, from bacteria to mammalian cell-culture systems are being used. Even though bacteria have some disadvantages, *Escherichia coli *is still one of the most commonly used organisms for the production of recombinant proteins [1-3].

The over-expression of recombinant proteins in bacteria often leads to their aggregation into protein deposits called inclusion bodies (IBs). However, recombinant protein production is stressful for the host bacterial cell, as the whole cell machinery has to adapt to the over-expression of foreign protein [4]. Therefore, the production process has to be carefully designed [5-7]. Extensive studies on bacterial IBs showed that if an overall friendlier production is used, a great proportion of properly folded and biologically active recombinant proteins are formed inside IBs [5-9]. Selection of the suitable production strain, optimization of the gene coding for the target protein, lowering the production temperature and careful design of medium composition are key factors in preparing IBs that will be composed of biologically active proteins [6,10]. Such IBs, which are made of biologically active proteins, are designated as non-classical IBs (ncIBs) [8,11].

Since IBs are highly pure protein deposit (over-expressed recombinant protein may represent up to 95% of total protein content [12]), ncIBs composed from active proteins are highly attractive to biotechnology and the developing field of nano-biotechnology [9,13,14]. Such ncIBs are highly attractive for downstream isolation of target proteins, as majority of other proteins can be simply washed from IBs after their isolation from bacterial cells [5,11]. In addition, IBs possessing active proteins can be used as active protein nanoparticles, with many possible applications [5,6,13,14].

However, in order to prepare quality active nanoparticles, both the protein production step (bacterial growth conditions) and the isolation process should be carefully optimized.

In the past, various mechanisms of bacterial cell disruption have been thoroughly studied. IBs can be isolated from bacterial cells using mechanical, chemical or biological methods for cell disruption [15]. However, the requirements for the liberation of ncIBs are different from those for the liberation of soluble proteins, or even classical IBs. NcIBs are composed from properly folded and biologically active proteins. Therefore such ncIBs can be used as active nanoparticles immediately after isolation process; the isolation step is thus very important.

Previous studies on ncIBs revealed that such IBs are more fragile compared to classical IBs and that they are even soluble in mild detergents (routinely used for washing of classical IBs) [8,11]. Therefore, classical washing procedures cause loss of target protein from ncIBs, so these have to be washed in low molar buffers (e.g. phosphate buffered saline (PBS), Tris/HCl buffer) or in pure water.

The isolation process should damage neither the ncIBs structure nor the quality of the protein trapped inside. It should enable liberation of pure ncIBs (with as little cell debris as possible) from bacterial cells so that the washing procedure should be limited to the minimum. For that reason we eliminated chemical lysis of bacterial cells [16-18], as the most often used detergents (e.g. N-lauroil sarcosine, TritonX 100, nonyl phenoxypolyethoxylethanol - NP-40) would (at least partially) dissolve ncIBs.

However, solubilisation of ncIBs can also be used to our advantage, as properly folded proteins trapped inside ncIBs can be extracted from ncIBs using mild detergents. Since the proteins are not denatured during the extraction process, no refolding is needed. While the denaturation/renaturation step is thus excluded, the protocol for downstream purification of target protein is simplified, more cost efficient and environment-friendlier [5,8,10,11,19].

Non-classical IBs are also very sensitive to the changes in buffer pH [20]: when transferred to low-pH buffer, they contract irreversibly. The contraction of IBs affects their sedimentation velocity during centrifugation, solubility, and above all the extraction of proteins.

The aim of the study was to find the most appropriate method for isolation of bacterial nanomaterial (ncIBs) composed from active proteins and, by maintaining intact both the structure of ncIBs and above all the structure of the proteins trapped inside ncIBs, to preserve their biological activity. Therefore, three different isolation methods were chosen and compared.

The first is a biological method that takes advantage of the enzyme lysozyme, which hydrolyzes the glycosidic bond in peptidoglycans found in the cell walls of bacteria. This is particularly true for Gram-positive bacteria. However Gram-negative bacteria are usually less sensitive to lysozyme treatment [21]. *E. coli *is a Gram-negative bacterium, and its cell wall is additionally surrounded by an outer membrane containing lipopolysaccharide (LPS), making it partially protected from lysozyme action. Nevertheless, as this method is frequently used for the isolation of proteins from *E. coli*, we decided to include it in our study. This method was chosen as no mechanical force is required to disrupt bacterial cells and we believe this enables isolation of the same form of IBs as they are inside the bacterial cell.

The other two investigated methods, homogenization and sonication, use mechanical forces to disrupt bacterial cells. During sonication the power of ultrasound is produced by the metal probe that oscillates with high frequency (20 kHz). This induces localized low pressure regions that induce shear stresses, which will result in cavitations of the bacterial cell wall, ultimately breaking the cells open [22]. The method is often used and recommended for bacterial cell disruption, as well as for protein isolation [23]. It is a simple and fast method for the isolation of small to medium sized samples. However, during sonication excessive heat can be generated, therefore the samples must be cooled during the isolation process.

On the other hand, high-pressure homogenization is the predominant method for bacterial cell disruption when moderate or large process volumes are in question [24]. The exact mechanism of cell disruption with high-pressure homogenization is still a matter of discussion. It is thought that internal and external forces produce tensions on the surface of the bacterial cell wall, which cause peptidoglicans to break and the formation of cavities in the cell wall [25,26]. The excessive generation of heat in the valve can represent a problem, however the effect can be diminished when samples are cooled prior to homogenization and are kept on ice during the isolation process.

## Results

### Isolation of inclusion bodies from bacterial cells

Three methods for bacterial cell disruption were compared to determine, which method is the most appropriate one for the isolation of IBs from bacterial cells. Two *E. coli *strains producing two structurally different proteins (granulocyte colony stimulating factor (G-CSF) and green fluorescent protein (GFP)) in the form of ncIBs were studied.

Following bacterial cell disruption, the soluble (SN) and insoluble cell fractions (pellet; P) were separated by centrifugation and analysed with SDS-PAGE. In case of G-CSF the presence of the target protein in each fraction was confirmed with immunoblot (Western blot). The soluble cell fraction is mainly composed from cytoplasmic proteins, while in the insoluble cell fraction IBs, membrane and cell wall fragments, as well as membrane bound proteins can be found.

**Figure 1:**
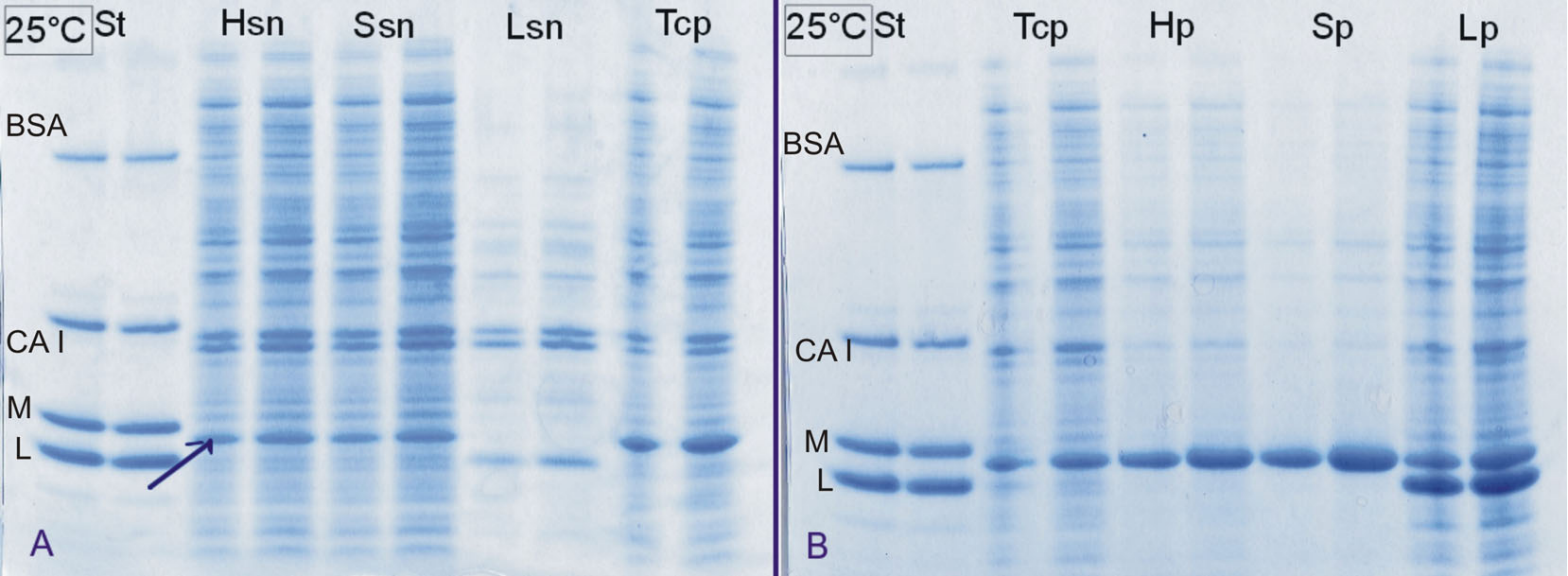
**SDS-PAGE analysis of proteins distributed between soluble and insoluble cell fraction after bacterial cell disruption (G-CSF)**. Target protein (G-CSF) was overexpressed in the form of ncIBs inside bacterial cells. Three mechanisms of bacterial cell disruption were studied: enzymatic lysis (L), high pressure homogenization (H) and sonication (S). After bacterial cell disruption soluble cell fraction (sn) and insoluble cell fraction (p) were separated with centrifugation and both samples were analysed with SDS-PAGE. Whole bacterial cells were also analysed (total cell proteins - Tcp). As standard (St) mixture of four proteins with known concentrations and known molecular weight was used. The arrow shows protein G-CSF. In the samples lysed with enzymatic lysis, the most abundant protein is enzyme Lysozyme. Legend: standard mixture (St) composition: BSA - Bovine serum albumin, 66.2 kDa (0.25 μg/gel). CA I - Carbonic anhydrase I, 31 kDa (0.5 μg/gel). M - Myoglobin, 17.2 kDa (1 μg/gel). L - Lysozyme, 14.4 kDa (1.5 μg/gel).

**Figure 2:**
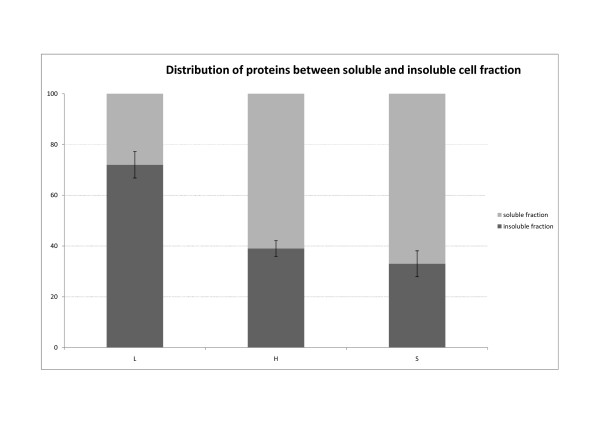
**Distribution of proteins between soluble and insoluble cell fraction**. After cell disruption densitometric analysis of SDS-PAGE was performed and the percentage of proteins in soluble vs. insoluble fraction was determined. Three mechanisms of bacterial cell disruption were studied: enzymatic lysis (L), high pressure homogenization (H) and sonication (S). After enzymatic lysis majority of the proteins were trapped in the insoluble fraction together with almost all recombinant protein G-CSF (Figure 1). However when the cells were disrupted with mechanical forces, more proteins were released to the soluble fraction.

**Figure 3:**
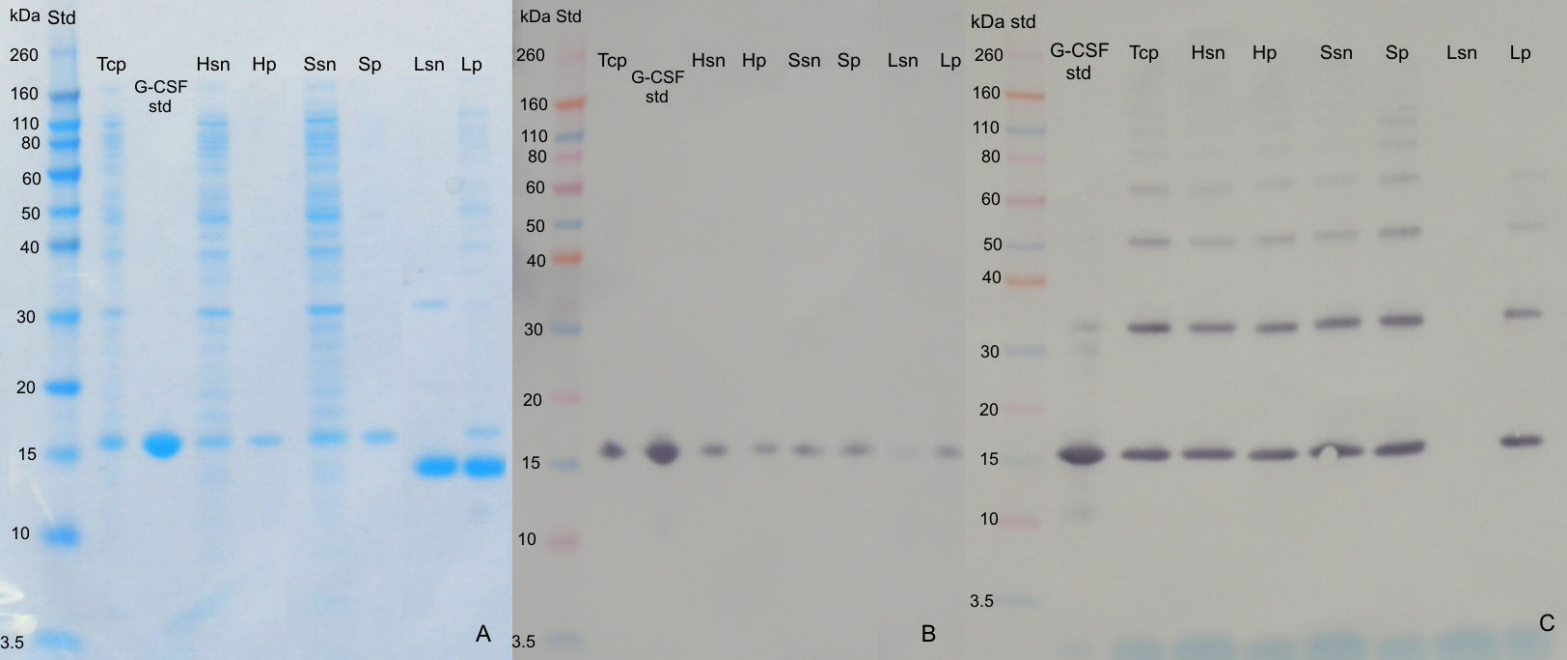
**SDS-PAGE analysis and western blot analysis of protein (G-CSF) distribution between soluble and insoluble cell fraction after bacterial cell disruption**. Target protein (G-CSF) was overexpressed in the form of ncIBs inside bacterial cells. Three mechanisms of bacterial cell disruption were studied: enzymatic lysis (L), high pressure homogenization (H) and sonication (S). After bacterial cell disruption soluble cell fraction (sn) and insoluble cell fraction (p) were separated with centrifugation and both samples were analysed with SDS-PAGE (A). Whole bacterial cells were also analysed (total cell proteins - Tcp). The most abundant protein in the samples lysed with enzymatic lysis is enzyme Lysozyme. Western blot analysis was also prepared to confirm the presence of target protein in individual fractions. Here two different analyses were prepared. The presence of protein in each fraction was confirmed with the western blot, where protein samples were reduced prior the analysis (B). Protein G-CSF is distributed between soluble and insoluble cell fraction when cells were disrupted with homogenization or sonication, whereas after enzymatic lysis almost all G-CSF is found in the insoluble cell fraction. The second western blot analysis was preformed with the protein samples without the addition of the reducing agent (C). Here the aggregation/polymerization of the protein G-CSF can be observed. Higher percentage of the aggregates can be observed in the insoluble sample (IBs) after sonication. Tcp - total cell proteins. Hsn - soluble cell fraction after homogenization. Hp - insoluble cell fraction after homogenization - Ibs. Ssn - soluble cell fraction after sonication. Sp - insoluble cell fraction after sonication - Ibs. Lsn - soluble cell fraction after enzymatic lysis. Lp - insoluble cell fraction after enzymatic lysis - Ibs.

**Figure 4:**
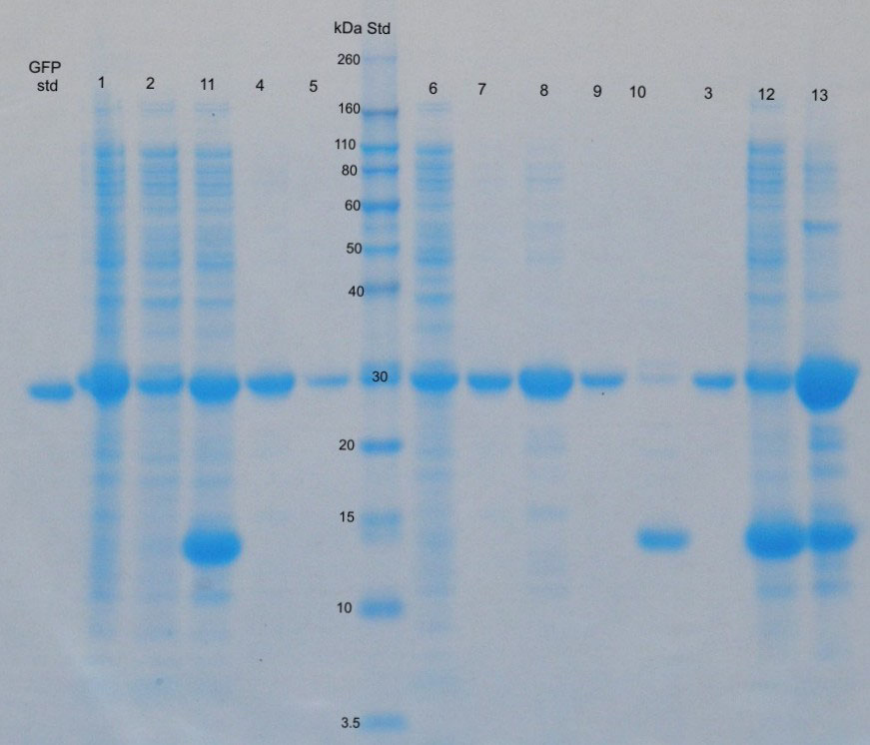
**SDS-PAGE analysis of protein (GFP) distribution between soluble and insoluble cell fraction after bacterial cell disruption**. Target protein (GFP) was overexpressed in the form of ncIBs inside bacterial cells. Three mechanisms of bacterial cell disruption were studied: high pressure homogenization (H), sonication (S) and enzymatic lysis (L). After bacterial cell disruption soluble cell fraction (sn) and insoluble cell fraction (p) were separated with centrifugation and both samples were analysed with SDS-PAGE. Whole bacterial cells were also analysed (total cell proteins - Tcp). After IBs isolation from the cells, they were solubilised in N-lauroul sarcosine (presented as sIBs). The insoluble residue (r) that remains after solubilisation of IBs is also presented. The most abundant protein in the samples lysed with enzymatic lysis is enzyme Lysozyme. 1 - total cell proteins. 2 - Hsn. 3 - Hp. 4 - HsIBs. 5 - Hr. 6 - Ssn. 7 - Sp. 8 - SsIBs. 9 - Sr. 10 - Lsn. 11 - Lp. 12- LsIBs. 13 - Lr.

The results are presented in Figures 1, 2, 3 and 4. SDS-PAGE analysis shows that the enzyme lysozyme sticks to (or possibly also enters into) the IBs, so it cannot be washed off (Figure 1, 3, 4). Densitometric analysis of SDS-PAGE shows that the percentage of proteins in the insoluble fraction is significantly higher when cells are disrupted by enzymatic lysis (more than 70% of total proteins) then by homogenization (39%) or sonication (33%) (Figure 2).

A difference can also be observed in the distribution of the target protein. G-CSF is present in both cell fractions (soluble and in insoluble), regardless of the cell disruption method. However, the amount of G-CSF in soluble fraction is significantly lower when cells are disrupted using enzymatic lysis, compared to mechanical methods (Figure 3). The same can be observed for protein GFP (Figure 4), where the majority of the GFP inside bacterial cells is actually trapped inside IBs. We believe that during enzymatic lysis the structure of ncIBs is not mechanically challenged, therefore it should remain the same as inside bacterial cells. No proteins are released from the ncIBs surface, as it seems to be the case with the mechanical methods. Since ncIBs are predominantly composed form the target protein (G-CSF or GFP), the most obvious is the difference in the distribution of these target proteins.

For more accurate analysis on G-CSF, Western blot analysis was performed (Figure 3b). Presence of G-CSF was confirmed in all samples. The significantly low amount of G-CSF in the soluble fraction after enzymatic lysis was also confirmed. Furthermore, to analyse the purity of the G-CSF in the samples, further analysis was performed, where protein samples were not reduced, thus some additional information about the purity of the G-CSF (formation of protein aggregates) was gathered (Figure 3c). In the sample lysed with lysozyme, very low amount of G-CSF dimmers can be observed (as in the undisturbed bacterial cells). On the other hand, it seems that both mechanical methods, homogenization and sonication, stimulates the polymerization (aggregation) of the monomer G-CSF protein and thus dimmers, trimmers, tetramers... are formed. Furthermore, it seems that sonication is even more damaging, as higher amounts of polymerization/aggregation can be observed in the samples (in soluble as well as in IBs fraction) after sonication (Figure 3c).

**Figure 5:**
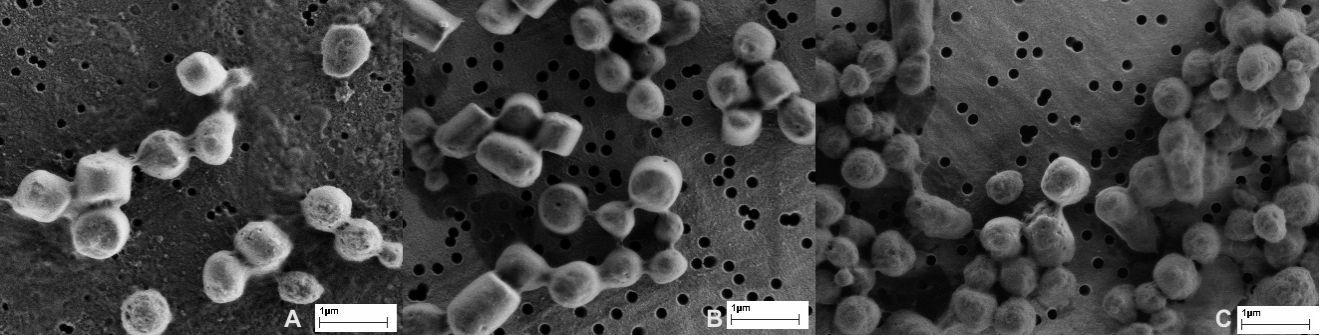
**Inclusion bodies after isolation from bacterial cells**. The figure shows G-CSF ncIBs observed under electron microscope after isolation from bacterial cells using three different methods of bacterial cell disrupion. IBs isolated with enzymatic lysis (A). IBs isolated with high-pressure homogenization (B). IBs isolated with ultrasound - sonication (C). When cells are isolated with enzymatic lysis, impurities can be observed, covering the gold-coated polycarbonate Isopore™ membrane filter. Most of the pores on the filter are also covered (A). On the other hand, when mechanical methods are used for IBs isolation, the pores on the filter are clearly visible and only some darker shadows (impurities) can be observed on the filter (B and C).

The effectiveness of IBs isolation was also observed under electron microscope (Figure 5). After enzymatic lysis impurities could be observed on the sample that could not be washed off with pure water (Figure 5a). On the other hand, IBs obtained after sonication or homogenization have fewer impurities (Figure 5b and 5c).

**Figure 6:**
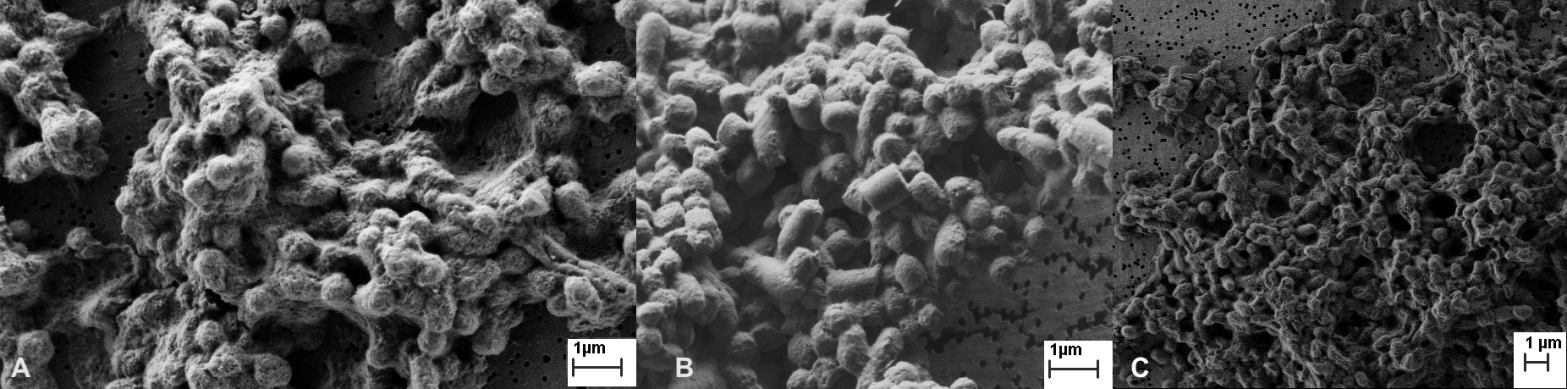
**Agglomerates of G-CSF IBs and cell debris after isolation**. IBs often form agglomerates together with the remains of the bacterial cells after enzymatic lysis (A). Similar agglomerates can also be observed after homogenization (B) and sonication (C) when pH of the buffer, where the cells are resuspended, is very low (pH 2-4). However, after enzymatic lysis more amorphous matter can be observed between the IBs in the agglomerate compared to IBs after mechanical isolation methods. This amorphous material is probably bacterial cell debris together with the enzyme lysozyme.

In addition, after enzymatic lysis parts of the bacterial cell wall and IBs were often observed to form agglomerates (Figure 6). Similar agglomerates could also be observed after sonication and homogenization, when the pH of the buffer in which cells are resuspended is very low (pH 2-4).

**Figure 7:**
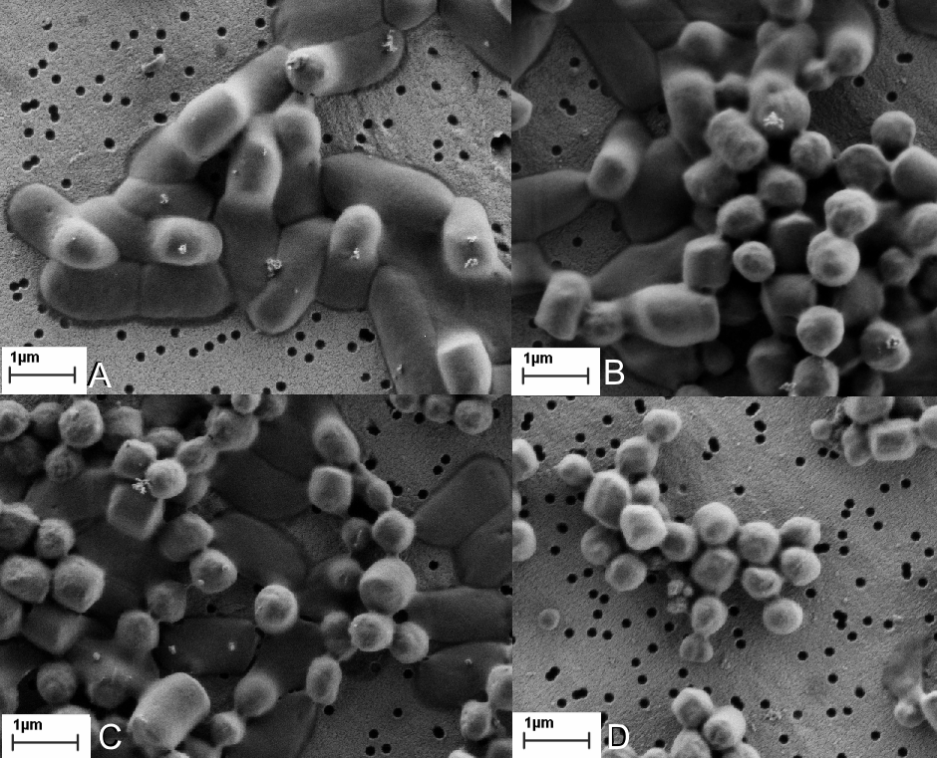
**Sonication of bacterial cells in time**. Whole bacterial cells producing G-CSF ncIBs before sonication. The outlines of ncIBs inside bacterial cells are visible (A). After 2 minutes of sonication the cell walls are disrupted, however ncIBs are still trapped inside the cells (B). Some ncIBs are isolated from the cells, while the others still remains trapped inside after 5 minutes of active sonication (C). All ncIBs are isolated and bacterial cell debris is removed from the sample after 8 minutes of sonication (D).

In the following step we determined the optimal conditions for IBs isolation using high-pressure homogenization and ultrasound (sonication). For a successful isolation of IBs using ultrasound, the optimal power was determined to be at 40%. At lower power bacterial cells did not break and IBs could not be isolated. For both investigated types of IBs (G-CSF and GFP) the optimal sonication time was 8 minutes. As it can be observed on Figure 7, although bacterial cells were already damaged after 2 minutes of sonication (Figure 7b), the IBs could not be isolated and lots of impurities were also present in the samples (after 5 minutes - Figure 7c). However the IBs sample appeared completely clear of bacterial cells and cell debris after 8 minutes (Figure 7d).

**Figure 8:**
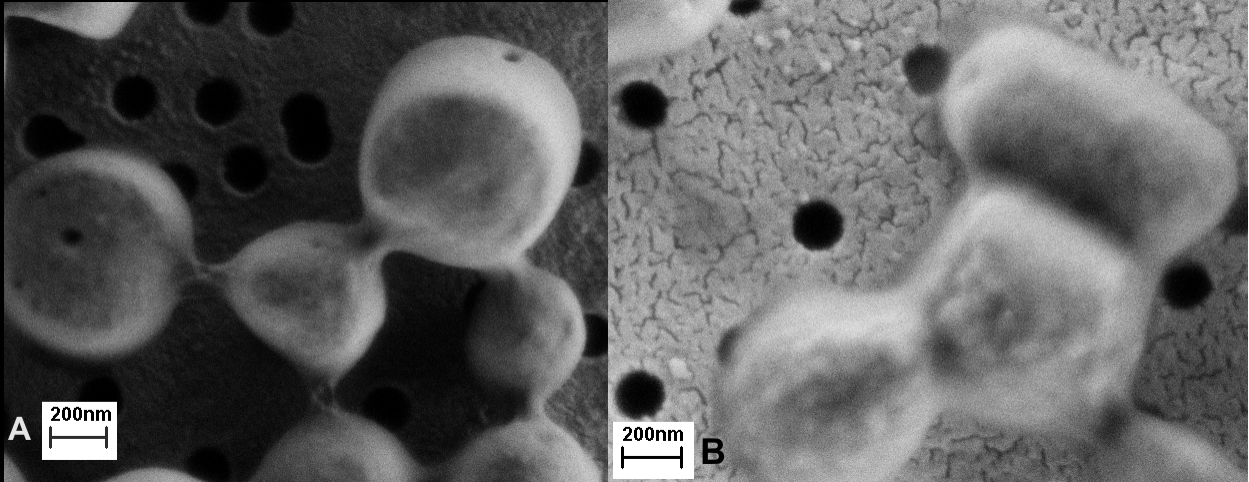
**G-CSF and GFP ncIBs after homogenization**. Bacterial cells were homogenised using high-pressure homogenizer. For G-CSF ncIBs 4 passages were run (A), whereas for GFP ncIBs 5 passages have to be run to completely remove bacterial cell debris from ncIBs (B).

For the successful isolation of IBs using high-pressure, the working pressure has to be between 75 and 80 MPa. At least 4 passages were necessary for G-CSF extraction (Figure 8a), whereas for GFP the minimum of 5 passages had to be run (Figure 8b). After the first passage cells break and in general the majority of soluble proteins can be isolated after the first or second passage. However, for the isolation of pure IBs a minimum of 4 passages had to be run. But in the case of ncIBs isolation, where washing with detergents is not applicable, additional passages of homogenization provide isolation of pure ncIBs. An independent study of the isolation protocol for other types of proteins showed that the number of passages has to be optimised for each protein individually. For example, for the successful isolation of IBs of the truncated form of Lymphotoxin-alpha (dN19 LT-α) at least 8 passages had to be run (data not shown).

### Effects of homogenization and sonication on isolated IBs

Electron microscopy showed that both mechanical cell disruption methods, homogenization and sonication, cause some damage to the surface of IBs. The difference between them is that after sonication IBs appear to have a more porous structure (Figure 5c), whereas after homogenization even broken IBs could be observed (Figure 5b).

**Figure 9:**
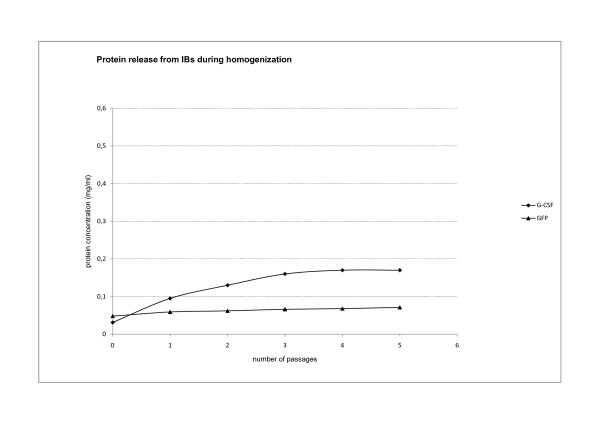
**Protein loss from ncIBs during homogenization**. NcIBs were isolated from bacterial cells using homogenization. They were thoroughly washed and resuspended in pure water. Isolated ncIBs were then treated as bacterial cells during homogenization. Five passages of high pressure homogenisation were run on GFP and G-CSF ncIBS to study the effect of homogenisation on protein release from the surface of ncIBs. After each passage proteins are lost from ncIBs. More proteins are lost from G-CSF ncIBs compared to GFP ncIBs.

**Figure 10:**
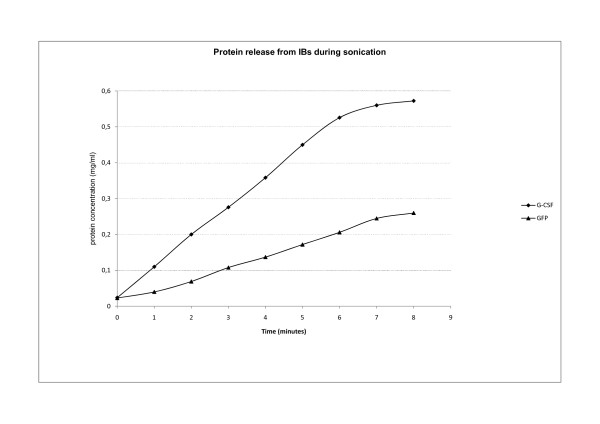
**Protein loss from IBs during sonication**. NcIBs were isolated from bacterial cells using homogenization. They were thoroughly washed and resuspended in pure water. Isolated ncIBs were then treated as bacterial cells during sonication. Aliquots were taken from the samples every minute. GFP and G-CSF ncIBS were studied. Proteins are lost from ncIBs during sonication. The quantity of protein loss during sonication is almost four times higher compared to high-pressure homogenization. As during homogenization also at sonication more proteins are lost from IBs composed from G-CSF.

In our study, IBs were isolated by homogenization and thoroughly washed, aliquoted and resuspended in pure water. "Crude" IBs isolated this way were then once again treated as the originating, intact bacterial cells during cell disruption. Two aliquots were made: one aliquot was homogenised with high-pressure homogenizer during five passages, while the other aliquot was sonicated for 8 minutes. Results are presented at Figures 9 and 10.

In both cases proteins were lost from the IBs into the solution. We found that more protein was lost from IBs during sonication. Amongst the proteins studied (G-GSF and GFP) more proteins were lost from G-CSF IBs both during homogenization and sonication.

**Figure 11:**
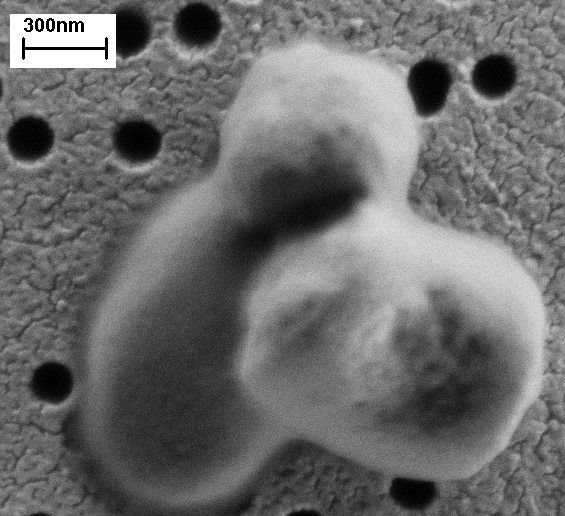
**GFP ncIBs after sonication with higher power output (50%)**. Isolated ncIBs were sonicated as in Figure 8, however higher power outputs were used. When sonication power output was higher (45% or more), non-classical IBs starts to disintegrate. Here ncIBs composed form GFP are presented. The structure of IBs is not compact anymore but seems softer, as if the ncIBs would melt.

Interestingly, if the power output during sonication was increased to 50% acoustic power (slight increase) GFP ncIBs would start decomposing and looked as if they were melting (Figure 11).

### Homogenization of isolated protein

The aim of the study was to determine not only the effect of the cell disruption method on the IBs, but above all to determine which method enables us to isolate active nanomaterial.

The effect of homogenization on biological activity was tested on a GFP protein mixture as well as on pure GFP and G-CSF extracted from IBs.

#### GFP

**Figure 12:**
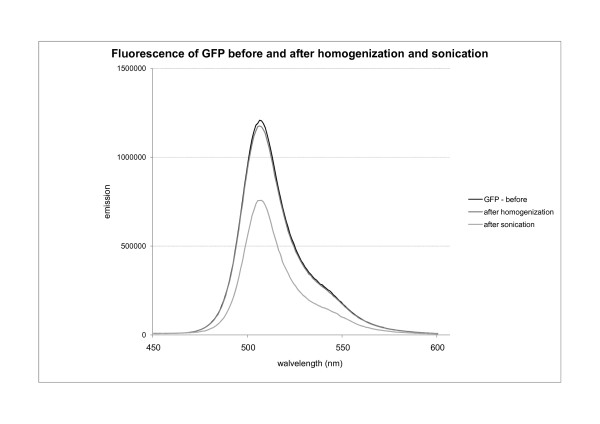
**The effect of homogenization and sonication on GFP activity (fluorescence)**. The fluorescence of GFP in protein mixture before and after homogenization was measured. GFP was extracted from IBs with N-lauroil sarcosine and remained in protein mixture of various proteins that compose IBs. Two aliquots of protein mixture were prepared and treated as described below: The first aliquot of the protein mixture was homogenized using high pressure homogenizer; five passages were made at the same conditions, usually used for IBs isolation from bacterial cells. The second aliquot of the protein mixture was sonicated with 40% power output at the same conditions, usually used for IBs isolation from bacterial cells.

The impact of homogenization on GFP biological activity (fluorescence) was tested. Fluorescence was measured prior to homogenization and thereafter each passage of homogenization. The results indicate that high-pressure homogenization may have no adverse effect neither on the fluorescence of pure GFP nor on the fluorescence of GFP in protein mixture (Figure 12).

#### G-CSF

The impact of homogenization on pure protein G-CSF was also tested. After several passages trough the valve, no aggregation or precipitation of the protein was noticed (no changes in protein concentration in the sample were detected).

### Sonication of isolated protein

Protein activity was not compromised during enzymatic lysis of bacterial cells and homogenization. The effects of ultrasound were analyzed on a protein mixture after extraction from inclusion bodies (GFP), as well as on pure proteins (GFP and G-CSF).

#### GFP

The impact of ultrasound on GFP activity was studied on pure protein as well as on protein extracted from IBs using mild extraction. When GFP was extracted from IBs, several other bacterial proteins were found in the sample. Protein activity (fluorescence) was measured prior to and after sonication.

Sonication at 40% output power had no effect on the activity (fluorescence) of pure GFP. However when a protein mixture was sonicated, the fluorescence of GFP after treatment was significantly weaker (Figure 12) with a reduction of 37% in fluorescence emission (biological activity of GFP).

#### G-CSF

**Figure 13:**
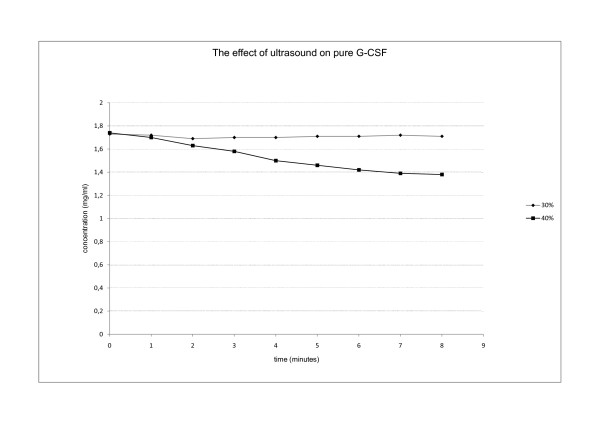
**The effect of ultrasound on pure G-CSF**. The effect of ultrasound on pure protein (G-CSF) was studied. At lower power output (30%) not strong enough to disturb bacterial cells no protein aggregation was noticed. However, at 40% power output (strong enough for IBs isolation) protein solution became cloudy as protein G-CSF started to precipitate from the solution. Protein concentration in protein solution was determined according to Bradford.

The effect of ultrasound was also tested on pure G-CSF protein. Sonication was tested at 30% and 40% power output. At 30% there was no effect on the protein, but such power output also does not cause bacterial cell disruption. On the other hand, a 40% power output enables bacterial cell disruption but also stimulates aggregation of pure G-CSF (Figure 13). The protein solution grew cloudy as the protein precipitated from the solution. In the beginning the aggregates were large enough and could be removed from the solution with centrifugation. However, prolonged time of sonication shattered aggregates into smaller particles and the solution remained cloudy, as the particles could not be removed by centrifugation.

## Discussion

Three methods of ncIBs isolation from bacterial cells were compared. Enzymatic lysis of the bacterial cell wall is very gentle towards ncIBs, since no mechanical forces are used. The two other methods studied (high-pressure homogenization and sonication with ultrasound) use mechanical force to break bacterial cell walls.

*E. coli *is a Gram-negative bacteria and therefore less sensitive to lysozyme. To achieve better results with the enzymatic lysis method, this is usually combined with one of the mechanical cell disruption methods or with freeze-thaw cycles. Since previously we already observed that ncIBs are very fragile and sensitive, we believe that mechanical methods may destroy their structure. Taking this into account, our goal was to optimize one non-mechanical method. Such method would enable mechanically undisturbed isolation of the ncIBs, which would maintain the same structure as inside bacterial cells. These undisturbed ncIBs could then be used as basis for the comparison of the degree of damage caused by different mechanical methods.

Results show that ncIBs can be isolated from *E. coli *using only enzymatic lysis, but some impurities (e.g. membranes, membrane bound proteins, cell wall fragments) still remain in the sample and cannot be washed off by simple washing (Figure 1, 3, 4 and 5). If the method is not optimized (e.g. time, enzyme quantity) the bacterial cell walls will crack, but the IBs will remain trapped inside bacterial cells. In addition, agglomerates of IBs, bacterial cells and cell debris will form (Figure 6). In samples where no agglomerates are formed, some of these impurities can be efficiently removed by electrophoretic deposition, a new method for separation of IBs from host bacteria and bacterial debris [27].

However, the method is not appropriate for the isolation of all proteins. Although lysozome is also active at lower temperatures (between 20 and 30°C) [28], the optimal temperature for lysozyme activity is around 35°C. If the protein in IBs is highly temperature sensitive, lysozyme can also be used at much lower temperatures (4°C), but then both the incubation time and the time of exposure of IBs to various external factors (e.g. buffer composition, bacterial enzymes...) has to be extended, and this could also damage the target protein.

Regardless of that, the results in Figure 1, 3 and 4 are much more alarming. The SDS-PAGE analysis of soluble and insoluble fractions after bacterial cell disruption show that even after extensive washing, large amounts of lysozyme remain in the insoluble fraction. This may be due to the enzyme adhering to the surface of IBs, or possibly even entering the pores.

It is known that as enzyme, lysozyme has remarkably high pI (over 11) with a net positive charge even well above neutral pH [29]. On the other hand our studies showed that IBs, bacterial cells and cell debris have a net negative charge in the neutral pH region [27]. One could assume that lysozyme "sticks" to the IBs and the remains of bacterial cells, acting as glue and binding them together. This greatly affects the stringency of the washing procedure, as if such ncIBs were employed as nanoparticles, the lysozyme in the sample would represent additional impurity. Therefore, in spite of the fact that amongst the methods tested this is the only one where the ncIBs structure and biological activity of the proteins inside ncIBs can be fully preserved, enzymatic lysis would not be our method of choice for ncIBs isolation.

Similar electrokinetic properties are also responsible for the formation of agglomerates during sonication and homogenization. In addition, as these formed only when buffers with low pH (under pH 4) were used (or used later for washing), we believe that this is due to specific electrokinetic properties of bacterial cell and IBs in the low pH region.

We confirmed this in a separate study, where electrokinetic properties of bacterial cells and IBs were investigated. This revealed that in the pH region between 2 and 4 the surface of bacterial cells is overall positively charged. On the other hand, the surface of IBs is on the whole negatively charged [27]. We believe that this difference in the electric potential causes the formation of agglomerates during homogenization and sonication in low pH buffers. When buffers with higher pH (4 - 9) are used, agglomerates do not form, as at pH above 4 both IBs and bacterial cells are overall negatively charged.

Chemical lysis is often described as a very successful method for IBs isolation [16]. In our case ncIBs are highly soluble in mild detergents and proteins are extracted from ncIBs already at very low detergent concentrations (0.2% NDSB, or N-lauroyl sarcosine) [5,8,11]. Therefore chemical lysis of the cell is not appropriate for isolation of ncIBs as it would solubilise them.

Our results demonstrate that high-pressure homogenization is easy to use for bacterial cell disruption. Importantly, various strains of *E. coli *show a different level of sensibility to high-pressure homogenization [26]. We have now shown that not only various strains, but also the same strain producing various recombinant proteins has an altered sensibility to high-pressure homogenization. This emphasizes the notion that the extraction method should be optimized for each protein separately.

NcIBs inside bacterial cells fill a significant part of the cell, and are not spheroid but rather rod-like shaped [7,20]. Electron microscopy revealed that after high-pressure homogenization ncIBs often have rough edges and appear broken (Figure 5). In particular, broken IBs appear in the sample when the bacterial culture is grown for 24 hours or more. Therefore, it seems that such rod-shape ncIBs are actually broken during homogenization. This can be avoided if cultivation time is shortened, as if ncIBs have less time to grow, fewer rod-like IBs will be present in the sample.

In addition, we show that only a negligible portion of proteins is lost from IBs during high-pressure homogenization. In order to get an idea of the scale of protein loss we took as maximal concentration (around 5 mg/ml) the amount of proteins that were extracted from IBs with 0.2% N-lauroyl sarcosine. As during high-pressure homogenization only up to 0.17 mg/ml of protein is lost in five passages, this represents approximately 3.5% of the total concentration.

In addition, studies on the effect of homogenization on the biological activity of GFP extracted from ncIBs showed that homogenization does not compromise the fluorescence of the protein. Therefore, we believe this method is suitable for a very efficient isolation of active ncIBs from bacterial cells.

Sonication was the third method studied. Here, no breakage of ncIBs could be observed under the electron microscope (Figure 5 and 7), but after sonication a higher protein concentration was detected in the soluble cell fraction (Figure 1, 2, 3 and 4) in comparison to homogenization. Furthermore, the surface of ncIBs appeared highly porous. Both results indicate that during sonication proteins are being lost from ncIBs in larger quantity. For example, the final protein concentration after 8 minutes of sonication reached only up to 0.58 mg/ml, which represents almost 12% of the maximal protein concentration extractable from G-CSF IBs (using 0.2% N-lauroyl sarcosine).

It is interesting to note that both during high-pressure homogenization as well as during sonication more G-CSF is lost from IBs in comparison to GFP. This is attributed to ncIBs structure. As our previous result show, there is a higher proportion of active protein inside G-CSF ncIBs (app. 50%) than in GFP ncIBs (app. 6%) [5,8,11]. We believe that properly folded proteins are less firmly bound within the ncIBs, thus they can be extracted from IBs with either mild detergents [20] or with mechanical forces like sonication or high-pressure homogenization.

Stathopulos and co-workers [30] reported that sonication of a range of structurally diverse proteins results in the formation of aggregates that have similarities to amyloid aggregates (sheet formation). This was confirmed also by our studies on protein activity. During sonication of pure protein G-CSF, aggregates were formed in the solution at 40% acoustic power output (conditions appropriate for bacterial cell disruption). If the time of sonication was prolonged, the big, flaky aggregates were broken into smaller aggregates that could not be easily centrifuged out of the solution. On the other hand, at lower acoustic power output no aggregates were detected. Previous studies on the chemical effects of ultrasound in aqueous solutions showed that due to high temperatures and pressures, gas bubbles are formed in the solution. At the air-water interface of the bubbles free radicals are generated, which in combination with high local temperatures and high local shear forces can react with other molecules [31] and induce sheet formation of proteins [32].

Studies on protein activity were performed on GFP, where the fluorescence of the protein was taken as an indicator of biological activity. Two samples of GFP were studied. In the first sample protein was extracted from IBs and purified. The activity of pure GFP remained unchanged after several successive cycles of sonication. The second sample consisted of GFP extracted from IBs, which contained also other bacterial proteins and unfolded molecules of GFP. After sonication the activity of the non-purified GFP in the solution was reduced by almost 40% compared to the activity of the protein prior to sonication. We believe that unfolded proteins and other impurities in the solution, combined with the effect of sonication, do affect the structure of properly folded proteins, decreasing the biological activity of the protein. Since similar protein composition can also be found inside ncIBs we believe, that sonication can affect also the biological activity of the proteins inside the ncIBs.

## Conclusions

Enzymatic lysis as well as two mechanical methods, high-pressure homogenization and sonication, were compared in the study.

Enzymatic lysis was determined as the method that is most gentle towards ncIBs since no mechanical forces that would damage the surface of ncIBs or the biological activity of the target protein inside ncIBs, are employed. However, the enzyme lysozyme strongly attaches to the surface of ncIBs and it cannot be removed from ncIBs by simple washing. Other cell debris present in the sample is also hard to remove, which represents additional impurity. Therefore, this method is not appropriate for isolation of pure and active nanoparticles.

The second method tested was sonication. Using this method ncIBs were easily isolated and no impurities remain in the sample. However, the power of ultrasound employed to disrupt bacterial cells also damaged the structure of ncIBs, as proteins were lost (released) from the surface. Furthermore, studies performed on GFP revealed that sonication affects protein structure, so the proteins lose their biological activity. To conclde, although sonication may be an appropriate method for the isolation of classical IBs, it was found as inappropriate for the isolation of active nanoparticles.

The last method tested was homogenization using a high-pressure homogenizer. Although during bacterial cell disruption part of ncIBs were damaged (broken) the overall protein loss from ncIBs is negligible. Furthermore, our study has shown that homogenization has no negative effect on biological activity of the proteins. For these reasons we found homogenization as the most appropriate method of isolation of active nanoparticles (ncIBs) amongst the three methods tested.

Our results indicate that future studies should focus on the development of new, possibly also non-mechanical based approaches that would enable isolation of native, undisturbed active protein nanoparticles.

## Methods

### Strains and plasmids

Recombinant *E. coli *production strain BL21 (DE3) (Novagen) was used in this study along with expression plasmids pET3a, and pET19b (Novagen), coding two structurally different proteins.

Details about the production of the BL21(DE3) [pET3a/P-Fopt5] strain for G-CSF production were described by Jevsevar et al. [11].

Green fluorescent protein (GFP) from plasmid pGFP (Clontech) was amplified by PCR and subcloned into the pET19b plasmid between the restriction sites XhoI and NcoI. Plasmid pET19b-GFP was then transformed into the *E. coli *BL21(DE3) production strain [6].

### Medium

LBG/amp 100 medium: 10 g/l BBL Phytone Peptone (Becton Dickinson), 5 g/l Bacto Yeast extract (Becton Dickinson), 10 g/l NaCl (Sigma), 100 mg/l ampicillin (Sigma), and 2.5 g/l glucose (Sigma).

GYSP/amp 100 medium: 20 g/l BBL Phytone Peptone (Becton Dickinson), 5 g/l Bacto Yeast extract (Becton Dickinson), 10 g/l NaCl (Sigma), 10 g/l glucose (Sigma), trace elements (FeSO_4_7H_2_O (40 mg/l), CaCl_2_2H_2_O (40 mg/l), MnSO_4_nH_2_O (10 mg/l), AlCl_3_6H_2_O (10 mg/l), CoCl_2_6H_2_O (4 mg/l), ZnSO_4_7H_2_O (2 mg/l), NMoO_4_2H_2_O (2 mg/l), CuSO_4_5H_2_O (1 mg/l), H_3_BO_3_(0.5 mg/l)), and 100 mg/l ampicillin (Sigma).

Inducer: 0.4 mM IPTG (Sigma).

### Culture conditions

Initial bacterial inoculum was prepared in a shake flask culture and grown overnight at 25°C at 160 rpm on the LBG/amp 100 medium. It was then transferred to the GYSP/amp 100 medium and induced with IPTG (immediate induction). The shake flask cultures were incubated at 160 rpm and 25°C (Kühner linear shaker) until the culture reached the stationary phase. After production, the biomass was aliquoted, centrifuged, and the supernatant was discarded. The bacterial pellet (biomass) was stored for further analysis.

### Bacterial cell disruption - Isolation of inclusion bodies

Bacterial pallet was resuspended in 10 mM TRIS/HCl buffer (pH8) in the ratio 4 ml of buffer to 1 g of wet biomass.

Three different cell disruption methods were tested.

#### Enzymatic lysis

Biomass was resuspened in 10 mM TRIS/HCl buffer (pH8) with addition of enzyme lysozyme (final concentration of lyzozyme - 2.5 mg/ml). The suspension was shaken for 30 minutes (60-75 rpm) at room temperature. Enzyme Benzonase (Merck) was added to the suspension (final concentration of Benzonase - 250 U/1 g of wet biomass). The suspension was shaken at room temperature for another half hour.

#### Sonication

Cells were disrupted using ultrasound in 500 W (20 kHz) Auto tune High Intensity Ultrasonc Processor ("sonicator"; Cole Parmer). Probe was submerged into the suspension with bacterial cells. Samples were sonicated for 10 minutes at 20 kHz using 1 s^-1 ^pulse and 30 - 40% acoustic power. The samples were cooled to 4°C prior to sonication and kept on ice during sonication, to prevent overheating.

#### High pressure homogenization

Bacterial cells resuspended in 10 mM TRIS/HCl buffer (pH8) were disrupted using high pressure homogenizer Emulsiflex-C5 (Avestin). Bacterial cells were disrupted at operating pressure 75 - 100 MPa; several passages were made. The samples were cooled to 4°C prior to homogenization and kept on ice during homogenization, to prevent overheating.

After cell disruption, the samples were observed under the microscope.

### Washing of inclusion bodies

The homogenate remaining after bacterial cell disruption was centrifuged at 10.000 g. The supernatant (soluble protein fraction; SN1) was stored for analysis. The pellet of IBs (P1) was briefly resuspended in pure water (washed) and centrifuged at 10.000 g. The washing procedure was repeated twice. Supernatants were discharged and IBs were used for further analysis.

### SDS-PAGE and Western blot analysis

Whole bacterial cells as well as samples after homogenization, sonication and enzymatic lysis (SN1, P1) were analyzed by SDS-PAGE. Nu-PAGE Bis-Tris gels (4-12%, Invitrogen) were used and stained with Colloidal Blue (Invitrogen). The calibration curve was prepared on each gel individually, with two samples of the same quantities of a protein mixture (0.25 μg bovine serum albumin, 0.5 μg carbonic anhydrase I, 1 μg myoglobin, 1.5 μg Lysozyme).

The presence of the target protein in each fraction was confirmed with immunoblot (Western blot). Proteins were transferred from Nu-PAGE Bis-Tris gels to the membrane Blot™ Dry Blotting System (Invitrogen). G-CSF was detected using rabbit anti hG-CSF primary antibodies in combination with goat horseradish peroxidase-linked secondary anti-rabbit antibodies. Horseradish peroxidase was than stained using 4-chloro-naphthol.

Quantity of the proteins was determined densitometrically using a ProExpress Imaging System (Perkin Elmer) with TotalLab 100 software. Samples from several independent experiments were analyzed.

### Extraction of properly folded proteins from inclusion bodies

IBs form bacterial cell isolated with high pressure solubilisation were used. Pellet of IBs was washed with pure water and resuspended with solubilizing buffer (40 mM Tris/HCl with 0.2% N-lauroyl sarcosine, pH 8.0) in ratio 1:40 [6,8,11]. The suspension was incubated for 24 hours at 20°C (Kühner shaker) and later centrifuged at 4400 g for 15 minutes. N-lauroyl sarcosine was removed from the sample during a one-hour incubation using a Dovex 1 × 4-50 ion exchange resin (Sigma). The protein mixture was then used for further experiments and for downstream protein purification.

### Downstream protein purification

Chromatographic separations were carried out on Knauer HPLC system equipped with two HPLC pumps (Knauer), a variable UV-Vis wavelength monitor (Knauer) and a Foxy Jr. (Teledyne ISCO) fraction collector.

#### G-CSF

The protein was isolated from the solubilized IBs. Technology for protein isolation was developed in our laboratory and previously described by V. Gaberc Porekar and V. Menart [19].

G-CSF was isolated on 2 ml column HR 2/10 (Amersham) packed with Ni-NTA Superflow (Qiagen). Sample was dissolved in 40 mM Tris/HCl (pH 8). A 20 mM Tris/HCl buffer containing 150 mM NaCl was used for washing the column and G-CSF was eluted using 20 mM acetate buffer containing 150 mM NaCl (pH 4). After isolation, the buffer was changed using Amicon Ultra-15 filter device (Millipore) with acetate buffer (pH 4) for further protein analysis.

#### GFP

The protein extracted from the IBs was further purified. The detailed protocol was described in our previous paper [6].

GFP was isolated on 10 ml column HR 10/100 (Amersham) packed with Chelating Sepharose Fast Flow medium (Amersham Biosciences) previously charged with Cu^2+^-ions. Sample was dissolved in a 100 mM K-phosphate buffer and applied to the column. A 100 mM K-phosphate buffer with 100 mM NaCl, pH 8.3, was used for washing the column and GFP was eluted with 100 mM imidazole in a 100 mM K-phosphate buffer with 100 mM NaCl, pH 8.3. After isolation, the buffer was changed using Amicon Ultra-15 filter device (Millipore) with phosphate buffered saline (PBS buffer) for further protein analysis.

### Sonication of isolated inclusion bodies

GFP and G-CSF inclusion bodies previously isolated from bacterial cell using high pressure homogenization were used in the study.

Isolated IBs were thoroughly washed with pure water. They were than resuspended in pure water. The samples were sonicated for 8 min at 20 kHz using 1 s^-1 ^pulse and 40% acoustic power. The samples were taken from the suspension during sonication after every minute of active sonication and used for further analysis. The samples were kept on ice during sonication, to prevent overheating.

Following sonication the samples were centrifuged at 9500 g for 10 minutes. The protein concentration in supernatant was determined according to Bradford method [33].

### Homogenization of isolated inclusion bodies

GFP and G-CSF IBs previously isolated from bacterial cell using high pressure homogenization were analysed in the study.

IBs were homogenised using high pressure homogenizer Emulsiflex-C5 (Avestin) at operating pressure 75 - 100 M Pa; five passages were made.

The samples were taken from the suspension after each homogenization passage and used for further analysis. The samples were kept on ice during homogenization, to prevent overheating.

Following homogenization the samples were centrifuged at 9500 g for 10 minutes. The protein concentration in supernatant was determined according to Bradford method [33].

### Sonication of isolated protein

Effect on ultrasound was tested on protein mixture after protein was extracted from inclusion bodies (GFP), as well as on pure protein (GFP and G-CSF).

#### GFP

The impact of ultrasound on pure GFP as well as on GFP extracted from IBs before purification was studied. Both protein samples were sonicated for 10 minutes in microcentrifuge tube at 20 kHz using 1 s^-1 ^pulse and 40% power with an appropriate probe. The tubes were kept in icy cold water during sonication, to prevent overheating. Protein activity (fluorescence) was measured before and immediately after sonication.

#### G-CSF

The impact of ultrasound on pure protein G-CSF was also studied. Probe was submerged into the buffer with pure protein. Samples were sonicated for 10 minutes at 20 kHz using 1 s^-1 ^pulse and 30 and 40% acoustic power independently. The samples were kept on ice during sonication to prevent overheating. The samples were taken from the suspension during sonication after each minute of active sonication and used for further analysis. Protein concentration in the sample was determined according to Bradford method [33].

### Homogenization of isolated protein

Effect of homogenization on protein biological activity was tested on pure GFP and G-CSF as well as on GFP in protein mixture. Protein GFP was extracted from inclusion bodies with N-lauroyl sarcosine (see chapter Extraction of properly folded proteins from IBs) and protein mixture was than homogenized using high pressure homogenizer Emulsiflex-C5 (Avestin) at operating pressure 75 - 100 M Pa; five passages were made. Pure protein was homogenised under the same conditions.

Samples were taken prior the homogenization and after each passage. Fluorescence of the GFP in the sample was analysed on QuantaMaster C-61 Spectrofluorometer (Photone Technology International).

Samples of pure G-CSF were centrifuged after the homogenization and protein concentration in the sample was determined according to Bradford method [33].

### Electron microscopy

IBs were thoroughly washed in pure water before observtion. Samples were prepared on a gold-coated polycarbonate Isopore™ membrane filter (filter pore size 0.22 μm) (Millipore). Samples were observed under a Zeiss SUPRA 35 VP electron microscope.

### Protein concentration analysis

Protein concentration in the supernatant after sonication and homogenisation of isolated IBs was determined according to Bradford [33] using UV-VIS spectrophotometer (Agilent 8453; Agilent technologies). Calibration curve was prepared with bovine serum albumin (BSA).

### Fluorescence

In the case of GFP, fluorescence was taken as a sign of correctly folded and oxidised protein. The fluorescence spectrum of GFP soluble in the cytoplasm (SN1) as well as of GFP extracted from IBs (SN2) was compared to the spectrum of in-house GFP standard isolated and purified in our laboratory.

Fluorescence of soluble GFP and GFP extracted from IBs with 0.2% N-lauroyl sarcosine (SN2) was measured on a QuantaMaster C-61 Spectrofluorometer (Photone Technology International).

## Competing interests

The authors declare that they have no competing interests.

## Authors' contributions

ŠP designed and performed all the experiments and prepared the manuscript as well as final data analysis and figures. RK was consolidating author and participated in the manuscript preparation. Both authors approved the final manuscript.
